# Physiological and Metabolic Effects of *Limnospira maxima* Inclusion in Fish Feed on the Liver, Intestine, and Fillet of Juvenile Nile Tilapia (*Oreochromis niloticus*)

**DOI:** 10.3390/ani16060889

**Published:** 2026-03-12

**Authors:** Layon Carvalho de Assis, Daniel Kurpan, Sílvia Pope de Araújo, Wassali Valadares de Sousa, Arthur Costa Santos, Bruna de Lemos Novo, Raphael de Oliveira Ribeiro, Carolina dos Santos Ferreira, Tatiana El-Bacha, Pedro Pierro Mendonça, Fábio César Sousa Nogueira, Alexandre Guedes Torres, Anita Ferreira do Valle

**Affiliations:** 1 Laboratório de Bioquímica e Biotecnologia de Algas, Departamento de Bioquímica, Instituto de Química, Universidade Federal do Rio de Janeiro, Av. Athos da Silveira Ramos 149, CT, Bloco A, sala 532A, Rio de Janeiro 21941-909, Brazil; layon@pos.iq.ufrj.br (L.C.d.A.); arthur.santos@gradu.iq.ufrj.br (A.C.S.); brunalemosnovo@iq.ufrj.br (B.d.L.N.); rapha.o.r@gradu.iq.ufrj.br (R.d.O.R.); avalle@iq.ufrj.br (A.F.d.V.); 2 Agroscope, Federal Department of Economic Affairs, Education and Research (EAER), 3003 Bern, Switzerland; 3 Departamento de Genética, Instituto de Biologia, Universidade Federal do Rio de Janeiro, Rio de Janeiro 21941-909, Brazil; wassali@ufrj.br; 4 Laboratório de Proteômica (LabProt) e Unidade Proteômica, LADETEC, Instituto de Química, Universidade Federal do Rio de Janeiro, Rio de Janeiro 21941-909, Brazil; fabiocsn@iq.ufrj.br; 5 LeBioME-Núcleo de Estudos com Bioativos, Mitocôndria, e Metabolismo da Placenta, Instituto de Nutrição Josué de Castro, Universidade Federal do Rio de Janeiro, Rio de Janeiro 21941-902, Braziltatiana@nutricao.ufrj.br (T.E.-B.); 6 Laboratório de Nutrição e Produção de Espécies Ornamentais, Instituto Federal do Espirito Santo, Campus Alegre, Postal 47, Alegre 29500-000, Brazil; ppmendonca@ifes.edu.br; 7 Centro de Pesquisa em Medicina de Precisão, Instituto de Biofísica Carlos Chagas Filho, Unidade Proteômica, Universidade Federal do Rio de Janeiro, Rio de Janeiro 21941-909, Brazil; 8 Laboratório de Bioquímica de Lipídeos e Lipidômica, Instituto de Química, Universidade Federal do Rio de Janeiro, Rio de Janeiro 21941-909, Brazil; torres@iq.ufrj.br

**Keywords:** aquaculture, sustainable aquaculture, microalgae, fatty acids, polyunsaturated fatty acids, dietary fat quality, proteomics

## Abstract

To reduce pressure on capture fisheries, sustainable aquaculture must decrease its dependency on fish meal and fish oil. Microalgae are a promising substitute due to their complete nutritional profile and low-footprint production process. This study examined the use of the cyanobacterium *Limnospira maxima* (commercially known as Spirulina) as a partial substitute for fish meal in feed for juvenile Nile tilapia (*Oreochromis niloticus*). We developed isoproteic (36%) and isoenergetic (3000 kcal kg^−1^) fish feed formulations containing 0% (control), 10%, 20%, 30%, or 40% *L. maxima* dry biomass. The experimental diets were then fed to 360 juvenile *O. niloticus* (1.32 ± 0.35 g) for 85 days using a randomized experimental design. The hepatic, intestinal, and muscle (fillet) tissues of the fish were collected for morphophysiological, fatty acid, and proteomic analyses. The intestinal coefficient, number of intestinal villi, villus height, and hepatosomatic index were essentially the same for all treatments (*p* > 0.05). Treatments containing 20–30% *L. maxima* exhibited a higher degree of unsaturation and better dietary fat quality. A greater abundance of the enzymes SOD, GSR, PRX1, and PLD3 in the experimental groups indicated higher antioxidant activity, whereas a greater abundance of acyl-CoA dehydrogenases indicated better use of fatty acids as an energy source. These trends were more evident in the 20–30% inclusion range. Thus, adding *L. maxima* to fish feed improves farming performance, fish health, and product quality. The results encourage the use of microalgae to promote more sustainable aquaculture.

## Simple Summary

Aquaculture is a fundamentally important sector of the global food supply, but it is also environmentally intensive. Conventionally used feed ingredients stand out among the factors that contribute to its high environmental footprint. For instance, fishmeal and fish oil rely on overexploited wild fisheries, while soybeans compete with human food resources. This study investigated how partially replacing fishmeal with the microalga Spirulina affects the morphophysiology and metabolism of Nile tilapia, one of the most widely farmed fish. In a previous study, we demonstrated that Spirulina improved the zootechnical performance of Nile tilapia. Here, we expand on those results by showing improvements in the fatty acid profiles and dietary fat quality of Spirulina-fed fish. Furthermore, proteomic analysis indicates a higher antioxidant capacity and more efficient lipid metabolism. Taken together, these results support the use of Spirulina as a fish feed ingredient to promote more sustainable aquaculture practices. Finally, this study provides a policy-based foundation for overcoming obstacles to implementing microalgae as an aquaculture feed ingredient.

## 1. Introduction

The global production of fisheries and aquaculture is projected to reach 205 million tons by 2032, a 10% increase relative to 2022. Most of this increase will come from aquaculture, which is expected to surpass 100 million tons in 2027 [1]. In 2022, aquaculture surpassed capture fisheries for the first time in aquatic animal production, reaching 94.4 million tons—that is, 51% of the global total. Of that production, 57% was destined for human consumption. Including fisheries, 89% of the total aquatic animal production was used for human consumption, while the remainder was used for non-food applications, primarily for fish meal and fish oil, the primary sources of protein and lipids in fish feed, respectively [1]. The aquaculture sector significantly impacts progress toward several United Nations Sustainable Development Goals (SDGs), including SDG 14 (Life Below Water). Thus, its technologies and practices have undergone substantial improvements in sustainability over the last 20 years. However, the sector still faces serious challenges that undermine its ability to achieve more sustainable outcomes. For example, despite advances in improving feed efficiency and reducing the use of marine resources, dependence on marine ingredients persists, and reliance on terrestrial ingredients has increased [2]. Research on new aquaculture feed ingredients has recently proliferated and is expected to continue expanding, supported by the rising demand for technologies that can replace fishmeal and fish oil in aquaculture feed [3].

Fish meal and fish oil provide essential amino acids (such as lysine and methionine) and fatty acids (such as eicosapentaenoic acid and docosahexaenoic acid) which are lacking in plant-based ingredients [4]. In this sense, microalgae stand out as a potential substitute ingredient. This diverse group of photosynthetic microorganisms has recently received attention from industry and academia due to its complete nutritional profile, wide array of bioactive compounds, and low-footprint production [5]. Cyanobacteria belonging to the genus *Limnospira* (commercially known as Spirulina) are the most widely produced and marketed microalgae in the world [6]. Their biomass may contain up to 70% protein, including all essential amino acids. Despite their low lipid content, they also contain essential polyunsaturated fatty acids (PUFAs) in their lipid compositions [7]. The incorporation of *Limnospira* spp. into fish feed has an overall positive effect on fish growth performance [8]. It has been shown to improve the flesh quality, pigmentation, and immune response of rainbow trout (*Oncorhynchus mykiss*); the reproductive performance of yellowtail cichlids (*Pseudotropheus acei*) and three-spot gouramis (*Trichopodus trichopterus*); and the tolerance of carp (*Cirrhinus carpio*) and catfish (*Clarias gariepinus* and *Clarias batrachus*) to heavy metals [9,10,11,12]. In tilapia farming, *Limnospira* spp. can promote growth and resistance to several pathogens, including *Aeromonas hydrophila* and *Vibrio algynolyticus* [13].

Tilapia (*Oreochromis* spp.) is an omnivorous fish that has been successfully farmed in over 90 countries, owing to its rapid and robust growth [14]. In 2023, it was the most widely produced fish in Brazil, making it the fourth largest producer in the world, behind China, Indonesia, and Egypt [1]. When cultivated, tilapia efficiently adapts to compound feed with a wide range of formulations [15]. Its compound feed is usually formulated containing relatively high concentrations of raw protein, especially in the early stages [16]. Despite being generally known for high feed efficiency and low fish-in:fish-out ratio, tilapia farming was outperformed by several other types of aquaculture in key performance indicators, such as feed use and wildlife impacts, in a recent review [17]. Therefore, a deeper understanding of the application of novel feed ingredients such as microalgae is crucial to further improve the sustainability of the tilapia farming value chain.

Given the global relevance of tilapia farming and the political and environmental pressure to adopt more sustainable aquaculture practices, this study examined the use of *Limnospira maxima* biomass as a partial substitute for fish meal in the feed of juvenile Nile tilapia (*Oreochromis niloticus*). Isoproteic and isoenergetic fish feed recipes were elaborated with increasing concentrations of *L. maxima* (up to 40%) and fed to juvenile *O. niloticus* in a randomized experimental design for 85 days. We then evaluated morphophysiological and metabolic outcomes in various tissues. The results presented here expand on the findings of a previous study on the zootechnical performance of the same experimental fish [18]. Taken together, these studies strongly support the inclusion of *L. maxima* in aquaculture to improve farming performance, fish health, and product quality.

## 2. Materials and Methods

### 2.1. Limnospira maxima Biomass Production

The cyanobacterium *L. maxima* (LEAF0045) was obtained from the Culture Collection of the Laboratory of Studies Applied to Photosynthesis (CMLEAF) at the Chemistry Institute of the Federal University of Rio de Janeiro in Brazil. The cells were cultivated in 80-L annular photobioreactors at 25 ± 2 °C and exposed to lateral illumination provided by fluorescent lamps (23 W, Phillips) under a 12:12 h photoperiod. The cultures were enriched with AO medium (pH 9.4) containing per liter: 13.61 g NaHCO_3_, 4.03 g Na_2_CO_3_, 0.50 g K_2_HPO_4_, 2.50 g NaNO_3_, 1.00 g K_2_SO_4_, 1.00 g NaCl, 0.20 g MgSO_4_·7H_2_O, 0.04 g CaCl_2_·2H_2_O, 0.01 g FeSO_4_·7H_2_O, 0.08 g Na_2_EDTA·2H_2_O, 0.05 mg cyanocobalamin, and 1.00 mL trace metal solution [19]. The biomass used for feed formulations (Table 1) was harvested during the exponential growth phase by filtration through a nylon membrane (100% NBC GIVE) and oven-dried at 37 ± 2 °C until it reached a constant weight.

### 2.2. Feed Preparation and Characterization

The experimental feed preparations contained soy bran, wheat bran, fish meal, corn meal, soybean oil, and dry *L. maxima* biomass at concentrations of 0% (control), 10%, 20%, 30%, and 40% (Table 2). Using the SuperCrac 6.1 Premium software, the recipes were elaborated to produce isoproteic (36% total protein) and isoenergetic (3000 kcal of digestible energy per kg) experimental diets. The software adjusts the concentration of each ingredient based on a categorical factor—here, *L. maxima* biomass—to achieve optimal formulations with no difference in proximate composition between treatments (Table 2). All dry ingredients were assembled and thoroughly homogenized with water at 60 °C. The resulting mixture was molded into 2-mm diameter pellets and dried at room temperature while sheltered from direct light until a constant weight was reached.

### 2.3. Experimental Design

We used a completely randomized experimental design that has been described elsewhere [18]. Briefly, 360 juvenile Nile tilapia (initial weight: 1.32 ± 0.35 g; initial length: 4.43 ± 0.42 cm) were divided into 20 60-L tanks, with 18 fish per tank, and fed the experimental diets four times daily (at 07:00, 09:00, 11:00, and 13:00 h) for 85 days. The physicochemical parameters of the water were monitored and maintained at 26–28 °C and pH 6.6–7.2. Dissolved oxygen and total ammonia concentrations ranged from 5.4 to 5.9 mg L^−1^ and 0.007 to 0.009 mg L^−1^, respectively [20]. Before the experiment, the fish were acclimated to the system and test routines for 7 days. At the end of the experiment, the fish were fasted for 24 h before the final biometric analyses were conducted. They were removed from the cultivation system and placed in an eugenol (25 mg L^−1^) alcoholic solution for anesthesia. The tilapia were slaughtered, and their livers, intestines, and fillets were immediately collected, measured, and stored at −80 °C until further analysis. The physiological parameters hepatosomatic index (HSI) and intestinal coefficient (IC) were calculated as follows:

HSI = (liver weight/body weight) × 100

IC = intestine length/body length

### 2.4. Histological Analysis

For optical microscopy, exerts from nonspecific regions of liver and the anterior intestine were fixed in formalin and then washed and dehydrated in an increasing concentration of ethanol. The samples were diaphanized in xylenes, immersed twice in paraffin at 60 °C for 30 min, and cooled to room temperature (20 ± 2 °C). The blocks were cut using a microtome (Leica, Wetzlar, Germany), and the sections were mounted on slides. The slides were dewaxed at 60 °C and stained with hematoxylin and eosin. After staining, the slides were mounted with transparent AcriLex glue and covered with a coverslip. Micrographs were taken using an optical microscope (Leica, Wetzlar, Germany) coupled with a computer containing Laz EZ v2.1.0 software (Leica, Wetzlar, Germany). The number and size of intestinal villi were determined manually.

### 2.5. Fatty Acid Profiles

Aliquots of 12.5 mg of lyophilized pooled samples of whole tissues were disrupted in methanol (0.5 mL) using ceramic beads on a FastPrep-24 bead mill (MP Biomedicals, Santa Clara, CA, USA) at 6 m s^−1^ for 1 min. This process was repeated eight times before adding 1.6 mL of methanol to achieve a sample:solvent ratio of around 6:1. The final solution underwent hydrolysis at 98 °C for 1 h after the addition of 0.4 mL hexane and 0.2 mL acetic chloride. Then, 2.0 mL of potassium carbonate solution (6% *m*/*v*) was added, and the mixture was centrifuged at 3000 rpm for 20 min at 24 °C. The resulting supernatant, which contained the fatty acid methyl esters (FAMEs), was collected.

The FAMEs were identified using gas chromatography coupled with a flame ionization detector (GC-FID; Agilent 7890A, Santa Clara, CA, USA) equipped with a fused silica capillary column (OMEGAWAX 320; 30 m × 0.32 mm × 25 µm; Supelco, Bellefonte, PA, USA). The data were processed using EZChrom Elite CDS A.04.10 software (Agilent Technologies Inc., Santa Clara, CA, USA). The injector and detector temperatures were fixed at 260 °C and 270 °C, respectively. The GC oven was operated at 120 °C, heated at a rate of 4 °C min^−1^ to 180 °C, held at the temperature for 1 min, and then heated at a rate of 3 °C min^−1^ to 210 °C and held for 15 min. The carrier gas (H_2_) flow was 2.13 mL min^−1^ with a 1:10 split. The flame gas flows were 25 mL min^−1^ N_2_, 30 mL min^−1^ H_2_, and 300 mL min^−1^ synthetic gas. The FAMEs were identified by comparing their retention times with those of a commercial FAME mix standard (GLC 463 Reference Standard, Nu-Check Prep. Inc., Elysian, MN, USA), and their concentrations (mg 100 g^−1^ dry weight) were calculated by peak area normalization after correcting the area with theoretical correction factors.

After characterizing the fatty acid profiles, we calculated the lipid quality indexes for the fish fillets as follows [21]:

AI = [C12:0 + (4 × C14:0) + C16:0]/(Σ MUFA + Σ ω-6 + Σ ω-3)

TI = (C14:0 + C16:0 + C18:0)/[(0.5 × Σ MUFA) + (0.5 × Σ ω-6) + (0.5 × Σ ω-3) + (0.5 × (Σ ω-3/Σ ω-6))]

h/H = (C18:1 ω-9 + C18:3 ω-6 + C18:3 ω-3 + C20:5 ω-3 + C22:6 ω-3)/(C14:0 + C16:0)

where AI is the atherogenic index, which indicates the correlation between the total saturated and unsaturated fatty acids; TI is the thrombogenic index; and h/H is the ratio of hypocholesterolemia and hypercholesterolemia. The fatty acid formulas are their concentrations in mg 100 g^−1^ of freeze dried tissue samples.

### 2.6. Proteomics

Proteins were extracted from tissue samples that were macerated in liquid nitrogen using a solution of 4% sodium dodecyl sulfate, 150 mmol L^−1^ Tris-HCl, and 1 mmol L^−1^ dithiotreitol. The mixtures were incubated in a Thermomixer (Eppendorf, Hamburg, Germany) at 300 rpm and 4 °C for 30 min. Then, they were ultrasonicated (Elmasonic S 60 H, Singer, Germany) for 10 min and centrifuged at 16,000× *g* and 4 °C for 20 min (Megafuge 8; Thermo Fisher, Waltham, MA, USA). The extracted proteins in the supernatant were precipitated in an acetone solution with 10% trichloroacetic acid at −30 °C for 16 h. The solution was then centrifuged at 16,000× *g* and 4 °C for 20 min, after which the pellets were washed twice with ice-cold acetone. The final pellets were dried on a SpeedVac.

The disulfide bridges in the protein samples were reduced by incubating them for 1 h at 35 °C in a 10 mmol L^−1^ dithiotreitol solution. The samples were then alkylated using iodoacetoamide (final concentration 40 mmol L^−1^) at room temperature for 30 min in the dark. After alkylation, the samples were diluted 1:8 in ammonium bicarbonate (100 mmol L^−1^). Trypsin was added at a 1:50 ratio to the mixture, which was incubated for 24 h at 28 °C. The enzymatic reaction was stopped by adding 1% trifluoroacetic acid. Two µg of the trypsinized samples were injected in an Easy-1000 nLC system (ThermoScientific, Waltham, MA, USA) coupled with a mass spectrometer Orbitrap Q-Exactive Plus (Thermo Scientific, Waltham, MA, USA). The trap column was a 20 mm HPLC Acclaim PepMap 100 C18 (3 µm and 75 µm spheres, Thermo Scientific, Waltham, MA, USA), and the analytical column was a 50 cm Easy-Spray (2 µm and 75 µm spheres, reverse phase, Thermo Scientific, Waltham, MA, USA). The main DDA-MS2 parameters were as follows: 17,500 resolution at *m*/*z* 200 Da, 1 × 10^6^ ion AGC target, maximum TI of 50 ms; *m*/*z* isolation window of 1.4, and minimum intensity threshold of 100,000 ions. The instrument was equipped with high-energy collision dissociation (HCD) using a normalized collision energy (NCE) of 30%.

The raw data were processed using FragPipe 21.0 software for protein identification, validation, and quantification (https://fragpipe.nesvilab.org/, accessed on 6 August 2024), using the MSFragger, Philosopher, and IonQuant tools. The identification was performed using a UniProt database for *O. niloticus* with 79,678 entries with common contaminants and decoys appended in Philosopher. The search parameters were set to allow for a maximum of two missed cleavages and to include only complete tryptic peptides. The following modifications were chosen: carbamidomethylation—fixed; methionine oxidation and acetylation (N-terminal protein)—variable. The precursor mass tolerance was set to 10 ppm and the ion fragment mass tolerance was set to 0.1 Da. The false discovery rate (FDR) was less than 1% at the peptide and PSM levels using the Philosopher. The FDR was less than 5% for qualitative proteomics and less than 1% for quantitative proteomics at the protein level. Precursor abundance was determined from ion intensities, and the matching between run (MBR) tool was used to improve ion identification in the samples, with a mass tolerance of 10 ppm and a maximum retention time to map features of 2 min. Label-free protein quantification (LFQ) was performed using the MaxLFQ method and the IonQuant algorithm with MBR and an FDR of 1%. The MBR was used with a maximum retention time shift of 10 min. Intensity was used for the LFQ.

### 2.7. Statistical Analysis

Each tank was considered an experimental unit. Each independent measurement was either an individual sample or a pooled sample from a tank. The effects of categorical factors on the results were analyzed in GraphPad Prism 10.2.3 by one-way analysis of variance (ANOVA), followed by Tukey’s honest significant difference test to determine whether there were significant differences between each individual treatment. The proteomic data were transformed using the binary logarithm and normalized using subtraction by the median before ANOVA and Tukey’s tests were performed. Before performing the ANOVA, the normality of the data was confirmed using the Shapiro–Wilk test. All statistical analyses were performed at a 95% confidence level (α = 0.05).

## 3. Results and Discussion

### 3.1. Morphophysiology of Oreochromis niloticus Liver and Intestine

Although using microalgae in fish feed offers great environmental benefits, it is crucial that this addition does not hinder fish development [22]. Despite the heterogeneous information reported in the literature regarding feed formulation, microalgae species and inclusion levels, and experimental designs and durations, it is widely accepted that microalgae can improve the zootechnical and physiological performance of fish [23]. For example, Youssef et al. [24] showed that the inclusion of up to 10% *Limnospira platensis* in *O. niloticus* feed for eight weeks improved intestinal immunity by increasing the height and width of intestinal villi, and the number of lymphocytes and goblet cells. Ibrahim et al., [25] added up to 3% of a microalgae mix containing *Limnospira* sp. to *O. niloticus* diets for 12 weeks, resulting in enhanced hepatic and digestive enzyme activities, immune response, and disease resistance.

Here, we extend our previous research on the zootechnical performance of juvenile Nile tilapia fed up to 40% *L. maxima*-based feed for 85 days [18] by presenting physiological and metabolic data. We found no significant differences in the IC, number of intestinal villi, villus height, and HSI between treatments (*p* > 0.05; Table 3). To improve the utilization of ingested food, fish can adapt their digestive tracts morphologically and physiologically depending on the offered diet [26]. As with many teleost fish, the anterior intestine of *O. nilotocus* is characterized by four distinct layers: mucosa, submucosa, muscularis, and serosa. The mucosa layer consists of a simple cylindrical epithelium with a brush border and goblet cells containing lamina with intraepithelial lymphocytes. The submucosa is formed by cells, collagen fibers, and blood vessels. The muscular layer consists of smooth muscles arranged in a circular pattern outside the serosa. This layer is characterized by connective tissue and pavimentous cells. These structures showed no difference in *O. niloticus* subjected to experimental diets with different levels of *L. maxima* biomass (Figure S1). This indicates that the addition of up to 40% *L. maxima* biomass to fish feed did not have a detrimental morphophysiological impact on their digestive tracts. It should be noted that the high variability observed in the number of villi and villus height, which is not uncommon in biological samples, suggests that these parameters should be further investigated in future research.

The fish liver performs several important functions, including the synthesis of certain amino acids, the production of plasma proteins, and protein deamination [27]. Excessive protein intake requires increased energy levels for metabolism, and the excess is stored as fat after deamination, which can overload liver function. By contrast, suboptimal protein intake leads to an energy demanding condition and causes oxidative stress. Both cases have a negative impact in growth and feed conversion [28,29,30,31]. Therefore, the liver can serve as an excellent indicator of nutritional constraints. Histological analyses of the hepatic parenchyma revealed hepatocytes with homogeneous cytoplasm and exocrine pancreatic acini dispersed throughout the tissue, with no observable differences between treatments (Figure S2). This indicates that the addition of up to 40% *L. maxima* biomass in fish feed did not have a detrimental morphophysiological impact on their livers.

### 3.2. Fatty Acid Profiles and Fillet Fat Quality

One of the main goals of using microalgae in fish production is to increase the content of unsaturated fatty acids in fish without relying heavily on fish oils. Many small fish are cultured using microalgae as live prey or by greenwater aquaculture in hatcheries [23]. In nature, fish PUFAs primarily originate from phytoplankton, which form the basis of the aquatic food web, since de novo PUFA synthesis in animals is limited [32]. For example, the fatty acid profile of the *L. maxima* biomass used in this study contained approximately 10% PUFAs, primarily linoleic acid, γ-linolenic acid, and α-linolenic acid (Table S2).

The main saturated fatty acid (SFA), MUFA, and PUFA in all tissues were palmitic acid (C16:0), oleic acid (C18:1*n*-9), and linoleic acid (C18:2*n*-6), respectively. In the liver, the highest concentrations of SFAs were found in the 40% *L. maxima* diet, while MUFAs and PUFAs were highest in fish fed 20% *L. maxima* (Table 4). Among the PUFAs, *n*-6 fatty acids were highest in the 20% treatment, whereas *n*-3 fatty acids were highest in the control group and decreased progressively with the increasing addition of *L. maxima* to the feed. The *n*-6/*n*-3 ratio was highest at 40% *L. maxima* inclusion. The intestinal tissue exhibited the highest concentrations of SFAs and PUFAs in the 20% treatment, and no difference in MUFA concentrations across treatments (Table 5). Similarly, the highest concentrations of *n*-6 and *n*-3 fatty acids were found in fish fed a 20% *L. maxima* diet. The *n*-6/*n*-3 ratio was essentially the same for all treatments. In the fillet, the highest concentrations of SFAs and MUFAs were found in the control group, whereas the highest concentrations of PUFAs were found in the 30% treatment, although this was not statistically different from the 20% and 40% treatments (Table 6). Similarly, no statistically significant differences were observed in the concentrations of *n*-3 fatty acid concentrations or *n*-6/*n*-3 ratios among treatments. Moreover, all tissues generally exhibited higher PUFA content in fish fed a 20–30% *L. maxima* diet. Interestingly, the same range of *L. maxima* addition resulted in optimal weight gain, growth, development, and survival rates for the fish used in this study [18] (Table 7).

The analysis of the liver, intestine, and fillet of *O. niloticus* fed increasing concentrations of *L. maxima* revealed different trends in fatty acid profiles in the different tissues. The selection of these tissues was based on the importance of obtaining a holistic overview of fatty acid metabolism, considering their widely different physiological roles in fish and other animals. For instance, the intestine is the primary site for lipid digestion and fatty acid absorption, muscle (fillet) is a major lipid storage tissue, and the liver is the central regulator of lipid metabolism. The wider variation of SFAs, PUFAs, and *n*-3 fatty acids in the liver when compared to the other tissues indicates its regulatory role in synthesizing, secreting, and degrading major lipids. In contrast, the fillet primarily increased the content of PUFAs, some of which are essential for fish physiology and have been shown to support cellular membranes, metabolic regulation, immune function, and steroid biosynthesis [33]. Different responses on *n*-3 and *n*-6 fatty acids and the *n*-6/*n*-3 ratio, which was only affected by diet in the liver, may be explained by the different activities of the elongases and desaturases that are responsible for PUFA synthesis. However, the activity of these enzymes is influenced not only by the tissue and the diet, but also by the environmental conditions and life stage of fish [34]. Therefore, more targeted research is needed to answer this question.

From a commercial standpoint, the degree of lipid unsaturation in fish fillets is of the utmost importance. High levels of unsaturation significantly affect the sensory properties of fillets by increasing their susceptibility to oxidation. This may reduce shelf-life and introduce negative off-flavors and odors [35]. On the other hand, from a consumer’s perspective, it is desirable to have more PUFAs in the fish fillets as a dietary protein source. Essential PUFAs, such as docosahexaenoic acid (DHA; C22:6*n*-3), for example, are important for brain and cardiovascular health, anti-inflammatory activity, and immune function [36]. Thus, the quality of dietary fat in *O. niloticus* fillets can be assessed based on their fatty acid profiles. In this study, we only examined indices of fat quality in fish fillets as a dietary protein source. Further research should be conducted to thoroughly investigate how different fatty acid profiles affect sensory properties and shelf-life.

**Figure 1:**
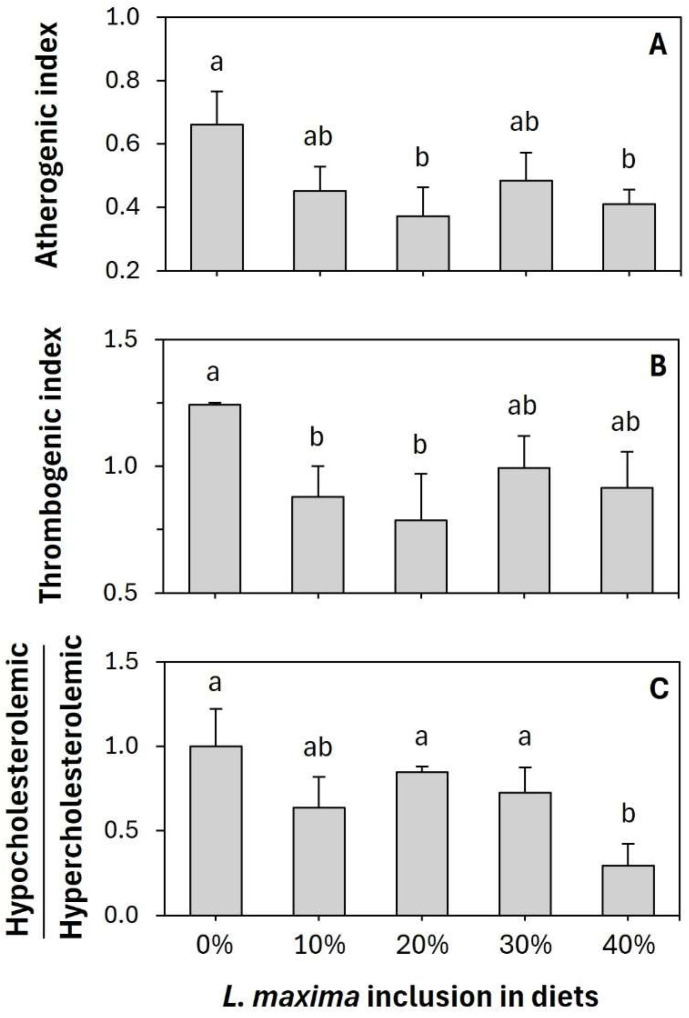
Dietary fat quality indices, atherogenic index (**A**), thrombogenic index (**B**), and H/h (**C**), of fillets of juvenile Nile tilapia (*Oreochromis niloticus*) fed experimental diets with increasing concentrations of *Limnospira maxima* biomass. The bars represent the means of three independent measurements, and the error bars are the standard deviations (±SD). Different alphabetic superscripts in a graph represent significantly different values, where a > b.

The atherogenic and the thrombogenic indices indicate the potential of a food to contribute to cardiovascular disease by comparing the levels of pro-atherogenic and thrombogenic saturated fatty acid levels to anti-atherogenic and thrombogenic unsaturated fatty acid levels [37]. Fillets from fish fed the experimental diet with a 20% *L. maxima* addition had the lowest AI and TI, significantly different from the control group (*p* < 0.05; Figure 1). Similarly, the hypocholesterolemic/hypercholesterolemic (h/H) index compares the content of unsaturated fatty acids, which lower cholesterolemia, with that of saturated fatty acids, which raise it, to evaluate the effect of dietary fatty acids on cholesterol metabolism. Fillets from fish fed the 40% *L. maxima* addition experimental diet had the lowest h/H index, which was statistically different from all other treatments except for the 10% treatment (Figure 1). A comprehensive comparison of the fatty acid profiles of the liver, intestine, and fillet, as well as the dietary fat quality indices of the fillets, showed that adding *L. maxima* to the diet of *O. niloticus* was potentially beneficial for both the farmed fish and their consumers. Still, it is essential to conduct sensorial analysis and acceptability tests to validate these results.

### 3.3. Proteomics, Enzymes, and Metabolism

**Figure 2:**
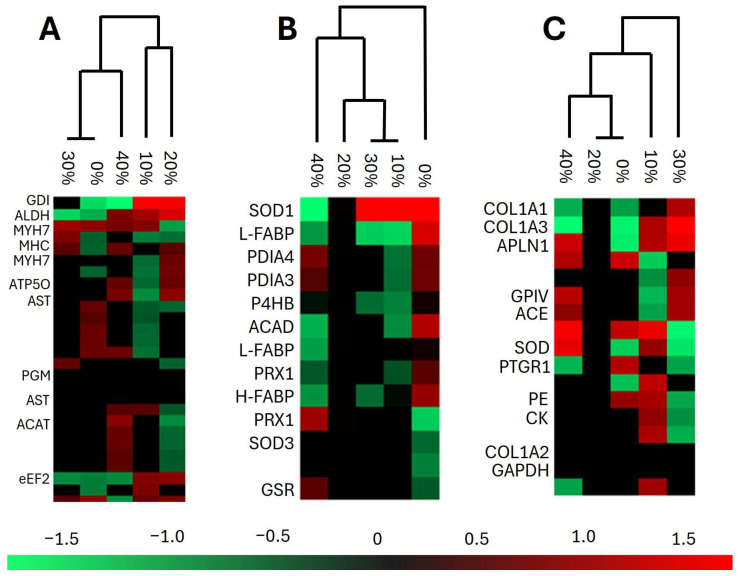
Protein abundance heatmaps of the fillet (**A**), liver (**B**), and intestine (**C**) of juvenile Nile tilapia (*Oreochromis niloticus*) fed experimental diets with increasing concentrations of *Limnospira maxima* biomass. Abbreviations: GDI = GDP dissociation inhibitor; ALDH = aldehyde dehydrogenase; MYH7 = myosin-7; MHC = myosin heavy chain; ATP5O = ATP synthase subunit O; AST = aspartate aminotransferase; PGM = phosphoglycerate mutase; ACAT = acetyl-CoA C-acetyltransferase; eEF2 = eukaryotic translation elongation factor 2; SOD = superoxide dismutase; FABP = fatty acid binding protein; PDIA = disulfide isomerase; ACAD = acetyl-CoA dehydrogenase; PRX = peroxiredoxin; GSR = glutathione-disulfide reductase; COL = collagen; APLN = aminopeptidase-like protein; GPIV = glycoprotein 4; ACE = angiotensin-converting enzyme; PTGR = prostaglandin reductase; PE = pancreatic elastase; CK = creatin kinase; GAPDH = glyceraldehyde-3-phosphate dehydrogenase. These heatmaps were optimized to highlight the proteins focused on this study.

Despite its massive potential in nutritional research, proteomic analysis remains underutilized in tilapia farming. The few available publications mostly relate to the exposition of fish to environmental stresses, or to the proteomic analysis of bacteria that have negative impacts on aquaculture [38,39,40]. None of the publications are related to tilapia nutrition. This study identified a total of 1758 proteins: 1463 in the liver, 408 in the intestine, and 689 in the fillet of juvenile Nile tilapia that were fed *L. maxima* (Table S2). Different concentrations of the microalga added to the experimental diets resulted in different protein patterns based on their relative abundance (Figure 2). One limitation of our investigation is that we did not carry out experimental validation of specific enzymes using methods such as Western blot or enzymatic activity assays. Instead, we relied on relatively robust proteomic analysis to gain an overall view of fish metabolism. Nevertheless, our results should be interpreted as indicative, and further specific analyses of enzymes of interest are encouraged. In line with the other results presented in this study, our assessment focused on antioxidant enzymes, and on enzymes related to fatty acid metabolism.

The antioxidant enzymes superoxide dismutase (SOD1 and SOD3), glutathione disulfide reductase (GSR), peroxiredoxin (PRX1), and phospholipase D3 (PLD3) were more abundant in the treatment groups that received *L. maxima*-containing feed than in the control group. Similarly, Hassaan et al. [41] observed increased SOD, catalase (CAT), and glutathione peroxidase (GSH-Px) activities in Nile tilapia supplemented with *Limnospira platensis* extract. Additionally, Ibrahim et al. [25] reported the upregulation of SOD, CAT, and GSH-Px at the gene expression level in Nile tilapia fed a microalgae mix containing *Limnospira* sp. The physiological role of GSR is to reduce glutathione disulfide and work with GSH-Px to combat free radicals in cells. PRX1 is abundant in the cytosol, where it reduces hydrogen peroxide and participates in cell division [42]. Moreover, PLD3 is a hepatoprotective enzyme, whose deficiency has been associated with hepatic inflammation in rats [43]. By contrast, disulfide isomerases (P4HB, PDIA3, and PDIA4) were less abundant in treatments containing *L. maxima* in the feed than in the control group. These enzymes regulate the activity of NADPH oxidase, which is a source of reactive oxygen species [44]. Therefore, these results indicate that fish fed a diet containing 20–30% *L. maxima* have increased resistance to oxidative stress.

The liver plays a central role in the synthesis, degradation, secretion, and storage of lipids [45]. Fatty acid-binding proteins (L-FABP, and H-FABP) were found in greater abundance in the control group and in the treatment with 40% *L. maxima* addition compared to the other treatments. These enzymes have multiple functions, including the ability to simultaneously bind to two molecules of long-chain fatty acid and to reduce the concentration of potentially toxic-free long-chain fatty acids [46,47]. It has been suggested that H-FABP influences intramuscular fat in pigs [48], which may explain the different trends in SFA concentrations in tissues as a function of *L. maxima* concentration in the investigated diets (Table 4, Table 5 and Table 6).

Beta-oxidation of saturated fatty acids mainly occurs in the liver and is catalyzed by three acyl-CoA dehydrogenase isoenzymes: very long-chain acyl-CoA dehydrogenase (VLCAD), medium-chain acyl-CoA dehydrogenase (MCAD), and short-chain acyl-CoA dehydrogenase (SCAD) [49]. This study found these enzymes to be in greater abundance in experimental diets containing *L. maxima* compared to the control group. These results align with the overall increase in lipid unsaturation observed in *O. niloticus* fillets (Table 6), although this trend was not evident in the fatty acid profiles of the liver and intestine (Table 4 and Table 5). In principle, the increased abundance of acyl-CoA dehydrogenases enables more efficient use of the lipid content in feed. This may explain the previously reported improvement in weight gain and feed conversion in fish fed *L. maxima*-containing feed [18].

### 3.4. Microalgae and the Future of Sustainable Aquaculture

It is generally accepted that the inclusion of microalgae in aquaculture feed can improve the sustainability and circularity of the sector. This is mainly due to (i) the partial or complete replacement of environmentally intensive ingredients, such as fishmeal and fish oil; (ii) the improvement of zootechnical performance in several fish species; and (iii) the improvement of meat quality, particularly with regard to unsaturated fatty acids [50]. In our previous work, we selected *L. maxima* as a feed ingredient over other microalgae species due to its growth performance and biochemical composition. We then demonstrated that the growth rate, survival rate, and weight gain of juvenile Nile tilapia were significantly increased with the addition of 20–30% *L. maxima* to their feed [18]. Here, we showed improvements in fatty acid quality with no morphophysiological impairment in the same fish, which, from a fish health perspective, encourages the use of *L. maxima* in fish feed. Today, however, there are two major constraints to implementing microalgae in the aquaculture sector: production capacity and price [51]. Even though cyanobacteria from the genus *Limnospira* (Spirulina) are the most widely produced microalga in the world, its production is still in the order of 10,000 tons, which is far below what is needed to supply the aquaculture sector [52]. For comparison, 4.4 million tons of Nile tilapia were produced in 2022 [53]. Moreover, the prices of microalgae are not yet competitive. The wholesale price of Spirulina is 5000–6000 USD per ton, whereas that of fishmeal, for example, is 1700–1800 USD per ton [54].

The future of microalgal biotechnology may hold great improvements, as recent policies and regulations encourage the use of algae as a renewable resource in all sectors, including aquaculture [55,56]. For instance, the European Commission recently identified the primary obstacles facing the European algae sector and is allocating resources to strengthen the sector in four key areas: (i) policy, environment, and regulations; (ii) finance and business development; (iii) consumers and value chains; and (iv) science, technology, and innovation [57]. The objective is to overcome the constraints in the production and pricing of algal biomass. In this sense, the results reported here contribute to strengthening area (iv) and promoting sustainable aquaculture in the future.

## 4. Conclusions

Morphophysiological and metabolic analyses of *O. niloticus* fed experimental diets containing up to 40% *L. maxima* confirmed the previously reported favorable zootechnical outcomes. The results presented here indicate that fish health and potential benefits for the final consumer in terms of meat quality were improved, particularly within the *L. maxima* concentration range of 20–30%. The partial substitution of fishmeal with *L. maxima* biomass is a way to increase the sustainability of the aquaculture sector and has no detrimental effects on the liver or intestine of the fish studied. Furthermore, the fatty acid profile and dietary fat quality indices of *O. niloticus* fillets improved significantly due to increased overall unsaturation levels and the presence of essential polyunsaturated fatty acids. Proteomic analyses focused on enzymes with antioxidant activity and related to fatty acid metabolism. Taken together, these results could explain the overall improvement in fish health and meat quality from a metabolic perspective.

## Figures and Tables

**Table 1:** Biochemical composition of *Limnospira maxima* dry biomass used for feed formulations.

Species	Components (%)			
	Proteins	Carbohydrates	Lipids	Ashes
*Limnospira maxima*	66.7 ± 4.00	1.74 ± 0.17	7.63 ± 1.87	11.7 ± 0.72

**Table 2:** Recipe and composition of the experimental diets containing different concentrations of *Limnospira maxima* biomass fed to juvenile Nile tilapia (*Oreochromis niloticus*). Data previously published in Araujo et al., 2024 [18]. Reproduced with permission from the publisher.

Ingredients (%)	*Limnospira maxima* Biomass Concentration in Diets				
	0%	10%	20%	30%	40%
Soy bran	64.75	52.00	39.25	26.50	13.75
Wheat bran	3.00	3.50	4.00	4.50	5.00
Fishmeal	10.00	8.75	7.50	6.25	5.00
Corn meal	20.25	23.00	25.75	28.50	31.25
Soybean oil	2.00	2.25	2.50	2.75	3.00
Sugar	0.00	0.50	1.00	1.50	2.00
*L. maxima*	0.00	10.00	20.00	30.00	40.00
**Composition (%)**					
Moisture	5.60	5.28	5.91	5.36	5.63
Crude lipids	2.08	1.71	1.23	1.83	3.39
Crude protein	36.1	36.5	36.0	37.0	37.3
Carbohydrates	4.36	4.01	4.85	4.60	4.42
Crude fiber	5.32	6.43	5.28	4.58	5.02
Ashes	7.47	7.38	6.06	5.35	5.12

There were no statistically significant differences between composition values among diets (*p* > 0.05).

**Table 3:** Physiological data (hepatosomatic index, intestinal coefficient, number and height of intestinal villi) of juvenile Nile tilapias (*Oreochromis niloticus*) fed a diet containing 0, 10, 20. 30, and 40% *Limnospira maxima* biomass.

Parameters	*Limnospira maxima* Biomass Concentration in Diets				
	0%	10%	20%	30%	40%
Intestinal coefficient	4.41 ± 0.18	4.89 ± 0.30	4.57 ± 0.24	4.85 ± 0.44	4.86 ± 0.17
Number of villi	58.0 ± 38.5	55.8 ± 41.1	55.0 ± 34.7	23.5 ± 18.1	80.3 ± 14.7
Villus height (µm)	152 ± 54.5	170 ± 43.3	109 ± 14.3	150 ± 34.8	142 ± 34.7
Hepatosomatic Index	1.99 ± 0.16	2.49 ± 0.72	2.21 ± 0.14	2.28 ± 0.39	2.33 ± 0.11

The data represent the mean ± standard deviation of four independent measurements. There were no significant differences for any parameter among the diets (*p* > 0.05).

**Table 4:** Fatty acid profile of liver tissue of juvenile Nile tilapias (*Oreochromis niloticus*) fed diets containing 0, 10, 20, 30, and 40% *Limnospira maxima* biomass.

Fatty Acid (mg 100 g^−1^)	*Limnospira maxima* Biomass Concentration in Diets				
	0%	10%	20%	30%	40%
*Saturated fatty acids (SFAs)*					
C16:0 (palmitic acid)	9.19 ± 1.15 ^ab^	8.33 ± 4.44 ^ab^	9.28 ± 1.53 ^ab^	5.18 ± 0.74 ^b^	13.1 ± 0.26 ^a^
C18:0 (stearic acid)	5.10 ± 0.50 ^ab^	3.19 ± 0.69 ^cd^	4.37 ± 0.66 ^bc^	2.21 ± 0.38 ^d^	5.88 ± 0.14 ^a^
Total SFAs	15.2 ± 1.75 ^ab^	12.8 ± 5.95 ^b^	15.5 ± 2.61 ^ab^	8.53 ± 1.32 ^b^	21.6 ± 0.72 ^a^
*Monounsaturated fatty acids (MUFAs)*					
C16:1*n*-7 (palmitoleic acid)	0.61 ± 0.21 ^a^	0.51 ± 0.13 ^a^	1.47 ± 0.88 ^a^	1.11 ± 0.55 ^a^	1.49 ± 0.55 ^a^
C18:1*n*-9 (oleic acid)	6.81 ± 1.27 ^a^	7.38 ± 2.78 ^a^	7.72 ± 1.01 ^a^	6.12 ± 0.01 ^a^	11.0 ± 3.43 ^a^
Total MUFAs	9.14 ± 1.64 ^a^	8.34 ± 2.08 ^a^	11.5 ± 2.00 ^a^	9.34 ± 1.79 ^a^	15.6 ± 5.14 ^a^
*Polyunsaturated fatty acids (PUFAs)*					
C18:2*n*-6 (linoleic acid)	5.07 ± 0.76 ^ab^	4.07 ± 0.69 ^bc^	6.41 ± 0.70 ^a^	3.53 ± 0.87 ^bc^	2.95 ± 0.65 ^c^
C18:3*n*-3 (α-linolenic acid)	0.22 ± 0.03 ^ab^	0.20 ± 0.04 ^ab^	0.29 ± 0.04 ^a^	0.14 ± 0.07 ^b^	0.09 ± 0.01 ^b^
C18:3*n*-6 (γ-linolenic acid)	0.36 ± 0.06 ^ab^	0.32 ± 0.18 ^ab^	0.67 ± 0.12 ^a^	0.40 ± 0.22 ^ab^	0.26 ± 0.02 ^b^
C20:2*n*-6 (eicosadienoic acid)	0.65 ± 0.06 ^a^	0.36 ± 0.25 ^ab^	0.64 ± 0.06 ^a^	0.33 ± 0.07 ^ab^	0.29 ± 0.05 ^b^
C20:3*n*-6 (eicosatrienoic acid)	0.37 ± 0.07 ^a^	0.35 ± 0.25 ^a^	0.71 ± 0.04 ^a^	0.41 ± 0.24 ^a^	0.24 ± 0.10 ^a^
C20:4*n*-6 (arachidonic acid)	2.20 ± 0.31 ^a^	2.19 ± 0.15 ^a^	1.96 ± 0.79 ^a^	0.95 ± 0.64 ^ab^	0.36 ± 0.13 ^b^
C22:3*n*-6 (dihomo-γ-linolenic acid)	0.43 ± 0.07 ^ab^	0.57 ± 0.06 ^a^	0.59 ± 0.16 ^a^	0.32 ± 0.19 ^ab^	0.15 ± 0.04 ^b^
C22:6*n*-3 (docosahexaenoic acid)	1.51 ± 0.09 ^a^	0.77 ± 0.68 ^ab^	1.04 ± 0.41 ^ab^	0.37 ± 0.20 ^ab^	0.15 ± 0.01 ^b^
Total PUFAs	10.6 ± 1.82 ^a^	7.68 ± 2.69 ^ab^	12.6 ± 1.02 ^a^	7.20 ± 3.14 ^ab^	4.64 ± 0.66 ^b^
Total *n*-3	2.00 ± 0.16 ^a^	1.60 ± 0.01 ^ab^	1.56 ± 0.49 ^ab^	0.73 ± 0.29 ^bc^	0.32 ± 0.06 ^c^
Total *n*-6	9.11 ± 1.15 ^ab^	8.26 ± 1.23 ^ab^	11.0 ± 0.56 ^a^	8.05 ± 1.20 ^b^	4.36 ± 0.59 ^c^
*n*-6/*n*-3 ratio	4.64 ± 0.27 ^b^	5.16 ± 0.77 ^b^	6.13 ± 0.18 ^b^	9.07 ± 1.99 ^ab^	13.5 ± 3.16 ^a^

The data represent the mean ± standard deviation of three independent measurements. Values in the same row with different alphabetic superscripts are significantly different (*p* < 0.05), where a > b > c > d.

**Table 5:** Fatty acid profile of intestinal tissue of juvenile Nile tilapias (*Oreochromis niloticus*) fed diets containing 0, 10, 20, 30, and 40% *Limnospira maxima* biomass.

Fatty Acid (mg 100 g^−1^)	*Limnospira maxima* Biomass Concentration in Diets				
	0%	10%	20%	30%	40%
*Saturated fatty acids (SFAs)*					
C16:0 (palmitic acid)	9.11 ± 4.09 ^a^	7.50 ± 0.56 ^a^	20.7 ± 10.9 ^a^	13.5 ± 1.54 ^a^	13.1 ± 1.21 ^a^
C18:0 (stearic acid)	8.95 ± 0.35 ^a^	4.65 ± 0.79 ^a^	7.22 ± 4.93 ^a^	7.43 ± 2.26 ^a^	6.00 ± 0.41 ^a^
Total SFAs	13.3 ± 0.42 ^b^	15.2 ± 1.22 ^b^	33.3 ± 10.4 ^a^	25.6 ± 3.80 ^ab^	23.2 ± 2.12 ^ab^
*Monounsaturated fatty acids (MUFAs)*					
C16:1*n*-7 (palmitoleic acid)	0.99 ± 0.34 ^a^	0.59 ± 0.10 ^a^	1.90 ± 1.07 ^a^	1.55 ± 0.38 ^a^	1.38 ± 0.59 ^a^
C18:1*n*-9 (oleic acid)	10.6 ± 4.48 ^a^	5.75 ± 1.55 ^a^	10.0 ± 2.01 ^a^	9.90 ± 2.61 ^a^	9.79 ± 2.22 ^a^
Total MUFAs	13.5 ± 4.74 ^a^	7.78 ± 1.56 ^a^	15.0 ± 1.90 ^a^	14.5 ± 3.68 ^a^	13.3 ± 2.76 ^a^
*Polyunsaturated fatty acids (PUFAs)*					
C18:2*n*-6 (linoleic acid)	3.29 ± 0.74 ^a^	2.98 ± 0.71 ^a^	4.78 ± 1.24 ^a^	1.87 ± 0.29 ^a^	1.92 ± 0.71 ^a^
C18:3*n*-3 (α-linolenic acid)	0.09 ± 0.03 ^a^	0.09 ± 0.04 ^a^	0.16 ± 0.09 ^a^	0.05 ± 0.01 ^a^	0.07 ± 0.01 ^a^
C18:3*n*-6 (γ-linolenic acid)	0.16 ± 0.04 ^a^	0.15 ± 0.06 ^a^	0.28 ± 0.11 ^a^	0.15 ± 0.02 ^a^	0.17 ± 0.06 ^a^
C20:2*n*-6 (eicosadienoic acid)	0.38 ± 0.12 ^a^	0.30 ± 0.09 ^a^	0.34 ± 0.13 ^a^	0.18 ± 0.04 ^a^	0.18 ± 0.06 ^a^
C20:3*n*-6 (eicosatrienoic acid)	0.30 ± 0.16 ^a^	0.25 ± 0.13 ^a^	0.25 ± 0.12 ^a^	0.13 ± 0.06 ^a^	0.26 ± 0.07 ^a^
C20:4*n*-6 (arachidonic acid)	0.61 ± 0.23 ^a^	0.77 ± 0.49 ^a^	0.69 ± 0.36 ^a^	0.23 ± 0.05 ^a^	0.30 ± 0.20 ^a^
C22:3*n*-6 (dihomo-γ-linolenic acid)	0.22 ± 0.09 ^a^	0.25 ± 0.16 ^a^	0.22 ± 0.06 ^a^	0.09 ± 0.03 ^a^	0.12 ± 0.05 ^a^
C22:6*n*-3 (docosahexaenoic acid)	0.21 ± 0.09 ^ab^	0.20 ± 0.11 ^ab^	0.40 ± 0.11 ^a^	0.12 ± 0.04 ^b^	0.20 ± 0.10 ^ab^
Total PUFAs	5.36 ± 0.97 ^ab^	4.80 ± 1.57 ^ab^	8.04 ± 1.99 ^a^	2.92 ± 0.33 ^b^	3.25 ± 1.09 ^b^
Total *n*-3	0.45 ± 0.06 ^ab^	0.37 ± 0.13 ^b^	0.74 ± 0.16 ^a^	0.23 ± 0.05 ^b^	0.33 ± 0.18 ^b^
Total *n*-6	4.91 ± 0.94 ^ab^	4.44 ± 1.46 ^ab^	7.29 ± 1.84 ^a^	2.69 ± 0.32 ^b^	2.93 ± 0.96 ^b^
*n*-6/*n*-3 ratio	10.9 ± 1.75 ^a^	12.4 ± 2.73 ^a^	9.76 ± 0.57 ^a^	12.0 ± 2.46 ^a^	10.0 ± 3.13 ^a^

The data represent the mean ± standard deviation of three independent measurements. Values in the same row with different alphabetic superscripts are significantly different (*p* < 0.05), where a > b.

**Table 6:** Fatty acid profile of fillet of juvenile Nile tilapias (*Oreochromis niloticus*) fed diets containing 0, 10, 20, 30, and 40% *Limnospira maxima* biomass.

Fatty Acid (mg 100 g^−1^)	*Limnospira maxima* Biomass Concentration in Diets				
	0%	10%	20%	30%	40%
*Saturated fatty acids (SFAs)*					
C16:0 (palmitic acid)	5.57 ± 0.90 ^a^	3.52 ± 0.31 ^bc^	2.36 ± 0.10 ^c^	4.54 ± 0.61 ^ab^	3.40 ± 0.27 ^bc^
C18:0 (stearic acid)	1.91 ± 0.14 ^a^	1.41 ± 0.01 ^b^	1.00 ± 0.05 ^c^	n.d.	1.42 ± 0.08 ^b^
Total SFAs	8.24 ± 1.13 ^a^	3.91 ± 1.38 ^b^	3.64 ± 0.13 ^b^	5.62 ± 1.57 ^ab^	5.15 ± 0.39 ^ab^
*Monounsaturated fatty acids (MUFAs)*					
C16:1*n*-7 (palmitoleic acid)	0.66 ± 0.13 ^a^	0.22 ± 0.03 ^bc^	0.14 ± 0.01 ^c^	0.42 ± 0.14 ^ab^	0.25 ± 0.05 ^bc^
C18:1*n*-9 (oleic acid)	6.16 ± 0.43 ^a^	2.58 ± 0.16 ^b^	1.63 ± 0.05 ^c^	3.39 ± 0.63 ^b^	2.62 ± 0.02 ^b^
Total MUFAs	7.52 ± 1.18 ^a^	3.28 ± 0.32 ^b^	3.62 ± 2.70 ^b^	4.30 ± 0.70 ^ab^	3.14 ± 0.50 ^b^
*Polyunsaturated fatty acids (PUFAs)*					
C18:2*n*-6 (linoleic acid)	2.59 ± 0.10 ^a^	1.68 ± 0.17 ^b^	1.55 ± 0.06 ^ab^	2.02 ± 0.15 ^ab^	1.68 ± 0.26 ^ab^
C18:3*n*-3 (α-linolenic acid)	n.d.	0.07 ± 0.02 ^a^	0.07 ± 0.01 ^a^	n.d.	0.07 ± 0.02 ^a^
C18:3*n*-6 (γ-linolenic acid)	0.12 ± 0.07 ^a^	0.07 ± 0.04 ^a^	0.15 ± 0.02 ^a^	0.20 ± 0.11 ^a^	0.20 ± 0.05 ^a^
C20:2*n*-6 (eicosadienoic acid)	n.d.	n.d.	0.17 ± 0.05 ^a^	0.18 ± 0.05 ^a^	0.15 ± 0.05 ^a^
C20:3*n*-6 (eicosatrienoic acid)	0.15 ± 0.09 ^a^	0.21 ± 0.12 ^a^	0.34 ± 0.01 ^a^	0.38 ± 0.01 ^a^	0.43 ± 0.02 ^a^
C20:4*n*-6 (arachidonic acid)	n.d.	n.d.	0.66 ± 0.06 ^a^	0.80 ± 0.03 ^a^	0.87 ± 0.01 ^a^
C22:3*n*-6 (dihomo-γ-linolenic acid)	n.d.	n.d.	0.22 ± 0.02 ^a^	0.32 ± 0.02 ^a^	0.32 ± 0.03 ^a^
C22:6*n*-3 (docosahexaenoic acid)	0.13 ± 0.02 ^b^	0.18 ± 0.01 ^a^	0.18 ± 0.02 ^a^	0.18 ± 0.02 ^a^	0.19 ± 0.01 ^a^
Total PUFAs	3.06 ± 0.46 ^c^	3.09 ± 0.16 ^bc^	3.38 ± 0.11 ^abc^	3.96 ± 0.15 ^a^	3.94 ± 0.45 ^ab^
Total *n*-3	0.17 ± 0.08 ^a^	0.24 ± 0.06 ^a^	0.30 ± 0.03 ^a^	0.25 ± 0.01 ^a^	0.25 ± 0.07 ^a^
Total *n*-6	2.90 ± 0.38 ^c^	3.01 ± 0.01 ^c^	3.09 ± 0.08 ^bc^	3.71 ± 0.10 ^a^	3.67 ± 0.27 ^ab^
*n*-6/*n*-3 ratio	18.7 ± 4.70 ^a^	13.4 ± 3.36 ^a^	10.7 ± 0.80 ^a^	15.1 ± 0.33 ^a^	15.9 ± 4.02 ^a^

The data represent the mean ± standard deviation of three independent measurements. Values in the same row with different alphabetic superscripts are significantly different (*p* < 0.05), where a > b > c. “n.d.” = not detected.

**Table 7:** Zootechnical performance of juvenile Nile tilapia (*Oreochromis niloticus*) fed a diet containing different levels of *Limnospira maxima* biomass for 85 days. Data previously published in Araújo et al. (2024) [18]. Reproduced with permission from the publisher.

Zootechnical Parameters	*Limnospira maxima* Biomass Concentration in Diets				
	0%	10%	20%	30%	40%
Weight (g)	22.0 ± 2.09 ^b^	32.1 ± 1.66 ^a^	35.5 ± 2.11 ^a^	31.2 ± 6.28 ^a^	29.8 ± 6.97 ^ab^
Weight gain (g)	20.7 ± 2.09 ^b^	30.8 ± 1.66 ^a^	34.2 ± 2.11 ^a^	29.9 ± 6.28 ^a^	28.5 ± 6.97 ^ab^
Survival rate (%)	75.0 ± 20.6 ^b^	77.8 ± 12.0 ^ab^	90.3 ± 6.99 ^ab^	93.1 ± 13.9 ^a^	77.8 ± 7.86 ^ab^
SGR (day^−1^)	3.27 ± 0.08 ^b^	3.70 ± 0.10 ^a^	3.84 ± 0.09 ^a^	3.65 ± 0.25 ^a^	3.57 ± 0.35 ^ab^
IFC (g)	34.6 ± 1.26 ^c^	48.0 ± 7.69 ^a^	44.2 ± 3.13 ^a^	41.6 ± 5.18 ^b^	41.0 ± 4.97 ^b^
AFC (g g^−1^)	1.80 ± 0.15 ^a^	1.77 ± 0.59 ^a^	1.39 ± 0.18 ^a^	1.57 ± 0.28 ^a^	1.77 ± 0.57 ^a^

The data represent the mean ± standard error of the mean of four independent measurements. Values in the same row with different alphabetic superscripts are significantly different, where a > b > c (*p* < 0.05). SGR = specific growth rate; IFC = individual feed consumption; AFC = apparent feed conversion.

## Data Availability

The original contributions presented in this study are included in the article/Supplementary Materials. Further inquiries can be directed to the corresponding author.

## Supplementary Materials

The following supporting information can be downloaded at: https://www.mdpi.com/article/10.3390/ani16060889/s1, Figure S1: Intestinal micrographs of juvenile Nile tilapia (*Oreochromis niloticus*) fed experimental diets with (A) 0%, (B) 10%, (C) 20%, (D) 30%, (E) 40% *Limnospira maxima* biomass. The height of intestinal villi is highlighted in yellow. The scale bars represent 200 µm.; Figure S2: Liver micrographs of juvenile Nile tilapia (*Oreochromis niloticus*) fed experimental diets with (A) 0%, (B) 10%, (C) 20%, (D) 30%, (E) 40% *Limnospira maxima* biomass. The arrows indicate vacuoles, AP = pancreatic acini, and H = hepatocyte. The scale bars represent 200 µm.; Table S1: Fatty acid profile of *Limnospira maxima* dry biomass used for experimental diets formulations. Data previously published in Araújo et al. (2024) [18]. Reproduced with permission from the publisher.; Table S2: Untreated output of proteomic analysis.

## Abbreviations

The following abbreviations are used in this manuscript:

SFA	Saturated fatty acid
MUFA	Monounsaturated fatty acid
PUFA	Polyunsaturated fatty acid
SOD	Superoxide dismutase
GSR	Glutathione disulfide reductase
PRX	Peroxiredoxin
PLD	Phospholipase
CAT	Catalase
GSH-Px	Glutathione peroxidase
PDI	Protein disulfide isomerase
FABP	Fatty acid binding protein
LCFA	Long-chain fatty acid
VLCAD	Very long chain acyl-CoA dehydrogenase
MCAD	Medium chain acyl-CoA dehydrogenase
SCAD	Short chain acyl-CoA dehydrogenase

## References

[1] FAO (2024). The State of World Fisheries and Aquaculture 2024.

[2] Naylor R.L., Hardy R.W., Buschmann A.H., Bush S.R., Cao L., Klinger D.H., Little D.C., Lubchenco J., Shumway S.E., Troell M. (2021). A 20-Year Retrospective Review of Global Aquaculture. Nature.

[3] Cottrell R.S., Blanchard J.L., Halpern B.S., Metian M., Froehlich H.E. (2020). Global Adoption of Novel Aquaculture Feeds Could Substantially Reduce Forage Fish Demand by 2030. Nat. Food.

[4] de Silva S.S., Anderson T.A. (1994). Fish Nutrition in Aquaculture.

[5] Acién F.G., Reis A., Wijffels R.H., Barbosa M., Verdelho V., Llamas B. (2021). The Role of Microalgae in the Bioeconomy. N. Biotechnol..

[6] Lafarga T., Fernández-Sevilla J.M., González-López C., Acién-Fernández F.G. (2020). Spirulina for the Food and Functional Food Industries. Food Res. Int..

[7] Damaciano S.F., Kurpan D., Ribeiro R.O., Torres I.B., França J.V.F., Barbarino E., Borges E.R., Perrone D., Valle A.F.d. (2024). Technological and Scientific Prospection of Phycocyanin Production from Spirulina (*Arthrospira* Spp.): Optimization and Application in Ice Cream. Braz. J. Food Technol..

[8] Zhang F., Man Y.B., Mo W.Y., Wong M.H. (2020). Application of Spirulina in Aquaculture: A Review on Wastewater Treatment and Fish Growth. Rev. Aquac..

[9] Sayed A.E.-D.H., El-Sayed Y.S., El-Far A.H. (2017). Hepatoprotective Efficacy of Spirulina Platensis against Lead-Induced Oxidative Stress and Genotoxicity in Catfish; Clarias Gariepinus. Ecotoxicol. Environ. Saf..

[10] Khanzadeh M., Esmaeili Fereidouni A., Seifi Berenjestanaki S. (2016). Effects of Partial Replacement of Fish Meal with Spirulina Platensis Meal in Practical Diets on Growth, Survival, Body Composition, and Reproductive Performance of Three-Spot Gourami (Trichopodus Trichopterus) (Pallas, 1770). Aquac. Int..

[11] Güroy B., Şahin İ., Mantoğlu S., Kayalı S. (2012). Spirulina as a Natural Carotenoid Source on Growth, Pigmentation and Reproductive Performance of Yellow Tail Cichlid Pseudotropheus Acei. Aquac. Int..

[12] Teimouri M., Yeganeh S., Amirkolaie A.K. (2016). The Effects of Spirulina Platensis Meal on Proximate Composition, Fatty Acid Profile and Lipid Peroxidation of Rainbow Trout (Oncorhynchus Mykiss) Muscle. Aquac. Nutr..

[13] Rosas V.T., Poersch L.H., Romano L.A., Tesser M.B. (2019). Feasibility of the Use of Spirulina in Aquaculture Diets. Rev. Aquac..

[14] Webster C.D., Lim C. (2024). Tilapia.

[15] Mjoun K., Rosentrater K., Brown M. (2010). Tilapia: Profile and Economic Importance. Fact Sheets. Paper 163. http://openprairie.sdstate.edu/extension_fact/163.

[16] Creswell D. (2005). The Feeding and Nutrition of the Tilapia: Part 2. Feed Management. Aquac. Asia Pac..

[17] Garlock T.M., Asche F., Anderson J.L., Eggert H., Anderson T.M., Che B., Chávez C.A., Chu J., Chukwuone N., Dey M.M. (2024). Environmental, Economic, and Social Sustainability in Aquaculture: The Aquaculture Performance Indicators. Nat. Commun..

[18] de Araújo S.P., de Assis L.C., Kurpan D., Telles M., de Carvalho A.G.A., Carneiro G.R.A., Nogueira F.C.S., Santos P., Barbarino E., Torres A.G. (2024). Screening Microalgae Strains for Fish Feed of Juvenile Nile Tilapia (*Oreochromis niloticus*) and Their Zootechnical Performance. J. Appl. Phycol..

[19] Andersen R., Andersen R. (2004). Algal Culturing Techniques.

[20] Beveridge M.C.M., McAndrew B.J., Beveridge M.C.M., McAndrew B.J. (2000). Tilapias: Biology and Exploitation.

[21] Moussavi Javardi M.S., Madani Z., Movahedi A., Karandish M., Abbasi B. (2020). The Correlation between Dietary Fat Quality Indices and Lipid Profile with Atherogenic Index of Plasma in Obese and Non-Obese Volunteers: A Cross-Sectional Descriptive-Analytic Case-Control Study. Lipids Health Dis..

[22] Vidotti R.M., Carneiro D.J., Viegas E. (2002). Growth Rate of Pacu, Piaractus Mesopotamicus, Fingerlings Fed Diets Containing Co-Dried Fish Silage as Replacement of Fish Meal. J. Appl. Aquac..

[23] Dineshbabu G., Goswami G., Kumar R., Sinha A., Das D. (2019). Microalgae–Nutritious, Sustainable Aqua- and Animal Feed Source. J. Funct. Foods.

[24] Youssef I.M.I., Saleh E.S.E., Tawfeek S.S., Abdel-Fadeel A.A.A., Abdel-Razik A.-R.H., Abdel-Daim A.S.A. (2023). Effect of Spirulina Platensis on Growth, Hematological, Biochemical, and Immunological Parameters of Nile Tilapia (*Oreochromis niloticus*). Trop. Anim. Health Prod..

[25] Ibrahim D., Abd El-Hamid M.I., Al-Zaban M.I., ElHady M., El-Azzouny M.M., ElFeky T.M., Al Sadik G.M., Samy O.M., Hamed T.A., Albalwe F.M. (2022). Impacts of Fortifying Nile Tilapia (*Oreochromis niloticus*) Diet with Different Strains of Microalgae on Its Performance, Fillet Quality and Disease Resistance to Aeromonas Hydrophila Considering the Interplay between Antioxidant and Inflammatory Response. Antioxidants.

[26] Honorato C.A., Almeida L.C., Nunes C.S., Carrilho E.N.V.M., Moraes G. (2014). Gastrointestinal Transit of Extruded or Pelletized Diets in Pacu Fed Distinct Inclusion Levels of Lipid and Carbohydrate. Pesqui. Agropecu. Bras..

[27] Honorato C.A., Assano M., Cruz C., Carneiro D.J., Machado M.R.F. (2013). Histologia Do Intestino de Tilapia Do Nilo Alimentados Com Dietas Contendo Diferentes Fontes de Proteína. Nucl. Anim..

[28] Talukdar A., Deo A.D., Sahu N.P., Sardar P., Aklakur M., Prakash S., Shamna N., Kumar S. (2020). Effects of Dietary Protein on Growth Performance, Nutrient Utilization, Digestive Enzymes and Physiological Status of Grey Mullet, *Mugil Cephalus* L. Fingerlings Reared in Inland Saline Water. Aquac. Nutr..

[29] Park I.-S., Lee S., Yoo G.-Y. (2022). Impact of Dietary Protein Levels on Growth, Feed Utilization, Body Composition, and Hematological Characteristics of Juvenile Hybrid Pufferfish (Takifugu Obscurus × T. Rubripes). Aquac. Rep..

[30] Guo W., Fu L., Wu Y., Liu H., Yang Y., Hu W., Xie S. (2021). Effects of Dietary Protein Levels on Growth and Feed Utilization in Non-Transgenic and Growth-Hormone-Gene Transgenic Common Carp (*Cyprinus Carpio* L.). Aquac. Rep..

[31] Yadata G.W., Ji K., Liang H., Ren M., Ge X., Yang Q. (2020). Effects of Dietary Protein Levels with Various Stocking Density on Growth Performance, Whole Body Composition, Plasma Parameters, Nitrogen Emission and Gene Expression Related to TOR Signaling of Juvenile Blunt Snout Bream (Megalobrama Ambylcephala). Aquaculture.

[32] Brett M., Muller-Navarra D. (1997). The Role of Highly Unsaturated Fatty Acids in Aquatic Foodweb Processes. Freshw. Biol..

[33] Zhang Z., Miar Y., Huyben D., Colombo S.M. (2024). Omega-3 Long-chain Polyunsaturated Fatty Acids in Atlantic Salmon: Functions, Requirements, Sources, de Novo Biosynthesis and Selective Breeding Strategies. Rev. Aquac..

[34] Zheng X., Torstensen B.E., Tocher D.R., Dick J.R., Henderson R.J., Bell J.G. (2005). Environmental and dietary influences on highly unsaturated fatty acid biosynthesis and expression of fatty acyl desaturase and elongase genes in liver of Atlantic salmon (*Salmo salar*). Biochim. Biophys. Acta.

[35] Ashton I.P., Allan Bremner H. (2002). Understanding lipid oxidation in fish. Safety and Quality Issues in Fish Processing.

[36] von Schacky C. (2021). Importance of EPA and DHA Blood Levels in Brain Structure and Function. Nutrients.

[37] Garaffo M.A., Vassallo-Agius R., Nengas Y., Lembo E., Rando R., Maisano R., Dugo G., Giuffrida D. (2011). Fatty Acids Profile, Atherogenic (IA) and Thrombogenic (IT) Health Lipid Indices, of Raw Roe of Blue Fin Tuna (*Thunnus thynnus* L.) and their salted product “Bottarga.”. Food Nutr. Sci..

[38] Pan J., Wang M., Zhu J., Huang Y., Zhang F., Li E., Qin J., Chen L., Wang X. (2024). Quantitative Proteomic and Metabolomic Profiling Reveals Different Osmoregulation Mechanisms of Tilapia Cells Coping with Different Hyperosmotic Stress. J. Proteom..

[39] Jia R., Hou Y., Feng W., Li B., Zhu J. (2022). Alterations at Biochemical, Proteomic and Transcriptomic Levels in Liver of Tilapia (*Oreochromis niloticus*) under Chronic Exposure to Environmentally Relevant Level of Glyphosate. Chemosphere.

[40] Li W., Su Y.-L., Mai Y.-Z., Li Y.-W., Mo Z.-Q., Li A.-X. (2014). Comparative Proteome Analysis of Two Streptococcus Agalactiae Strains from Cultured Tilapia with Different Virulence. Vet. Microbiol..

[41] Hassaan M.S., Mohammady E.Y., Soaudy M.R., Sabae S.A., Mahmoud A.M.A., El-Haroun E.R. (2021). Comparative Study on the Effect of Dietary β-Carotene and Phycocyanin Extracted from Spirulina Platensis on Immune-Oxidative Stress Biomarkers, Genes Expression and Intestinal Enzymes, Serum Biochemical in Nile Tilapia, *Oreochromis niloticus*. Fish Shellfish Immunol..

[42] Rhee S.G., Chae H.Z., Kim K. (2005). Peroxiredoxins: A Historical Overview and Speculative Preview of Novel Mechanisms and Emerging Concepts in Cell Signaling. Free Radic. Biol. Med..

[43] Gavin A.L., Huang D., Huber C., Mårtensson A., Tardif V., Skog P.D., Blane T.R., Thinnes T.C., Osborn K., Chong H.S. (2018). PLD3 and PLD4 Are Single-Stranded Acid Exonucleases That Regulate Endosomal Nucleic-Acid Sensing. Nat. Immunol..

[44] Janiszewski M., Lopes L.R., Carmo A.O., Pedro M.A., Brandes R.P., Santos C.X.C., Laurindo F.R.M. (2005). Regulation of NAD(P)H Oxidase by Associated Protein Disulfide Isomerase in Vascular Smooth Muscle Cells. J. Biol. Chem..

[45] Jia R., Cao L.-P., Du J.-L., He Q., Gu Z.-Y., Jeney G., Xu P., Yin G.-J. (2020). Effects of High-Fat Diet on Steatosis, Endoplasmic Reticulum Stress and Autophagy in Liver of Tilapia (*Oreochromis niloticus*). Front. Mar. Sci..

[46] Wang G., Bonkovsky H.L., de Lemos A., Burczynski F.J. (2015). Recent Insights into the Biological Functions of Liver Fatty Acid Binding Protein 1. J. Lipid Res..

[47] Lu Y.-C., Chang C.-C., Wang C.-P., Hung W.-C., Tsai I.-T., Tang W.-H., Wu C.-C., Wei C.-T., Chung F.-M., Lee Y.-J. (2020). Circulating Fatty Acid-Binding Protein 1 (FABP1) and Nonalcoholic Fatty Liver Disease in Patients with Type 2 Diabetes Mellitus. Int. J. Med. Sci..

[48] Gerbens F., de Koning D.J., Harders F.L., Meuwissen T.H., Janss L.L., Groenen M.A., Veerkamp J.H., Van Arendonk J.A., te Pas M.F. (2000). The Effect of Adipocyte and Heart Fatty Acid-Binding Protein Genes on Intramuscular Fat and Backfat Content in Meishan Crossbred Pigs. J. Anim. Sci..

[49] Lehninger A.L., Nelson D.L., Cox M.M. (2005). Lehninger Principles of Biochemistry.

[50] Yaakob Z., Ali E., Zainal A., Mohamad M., Takriff M.S. (2014). An Overview: Biomolecules from Microalgae for Animal Feed and Aquaculture. J. Biol. Res.-Thessalon..

[51] Madeira M.S., Cardoso C., Lopes P.A., Coelho D., Afonso C., Bandarra N.M., Prates J.A.M. (2017). Microalgae as Feed Ingredients for Livestock Production and Meat Quality: A Review. Livest. Sci..

[52] Girotto F., Schievano A., Idà A., Clerici G.R., Sala G., Goglio A., Kurpan D., Bombelli P., Toschi I., Bocchi S. (2022). Earthenware-Based Biofilter Configuration for Spirulina Cultivation on Nutrients Recycled from Food-Industry Waste Streams: A Preliminary Study. Bioresour. Technol. Rep..

[53] FAO (2022). The State of World Fisheries and Aquaculture 2022.

[54] FRED Global Price of Fish Meal. https://fred.stlouisfed.org/series/PFISHUSDA.

[55] (2021). Strategic Guidelines for a More Sustainable and Competitive EU Aquaculture for the Period 2021 to 2030.

[56] (2021). On a New Approach for a Sustainable Blue Economy in the EU Transforming the EU’s Blue Economy for a Sustainable Future.

[57] (2022). Towards a Strong and Sustainable EU Algae Sector.

