# Supplementary material for: Physiological and Metabolic Effects of Limnospira maxima Inclusion in Fish Feed on the Liver, Intestine, and Fillet of Juvenile Nile Tilapia (Oreochromis niloticus)
Source: Animals (Basel). 2026 Mar 12;16(6):889. doi: 10.3390/ani16060889 (PMC13023301; doi:10.3390/ani16060889)
Supplement: Supplementary file 1 [file animals-16-00889-s001.zip › Supplementary_material_REV.pdf]

animals

MDPI

Supplementary Material

Physiological and Metabolic Effects of *Limnospira maxima* Inclusion in Fish Feed on the Liver, Intestine, and Fillet of Juvenile Nile Tilapia (*Oreochromis niloticus*)

Supplementary Figures

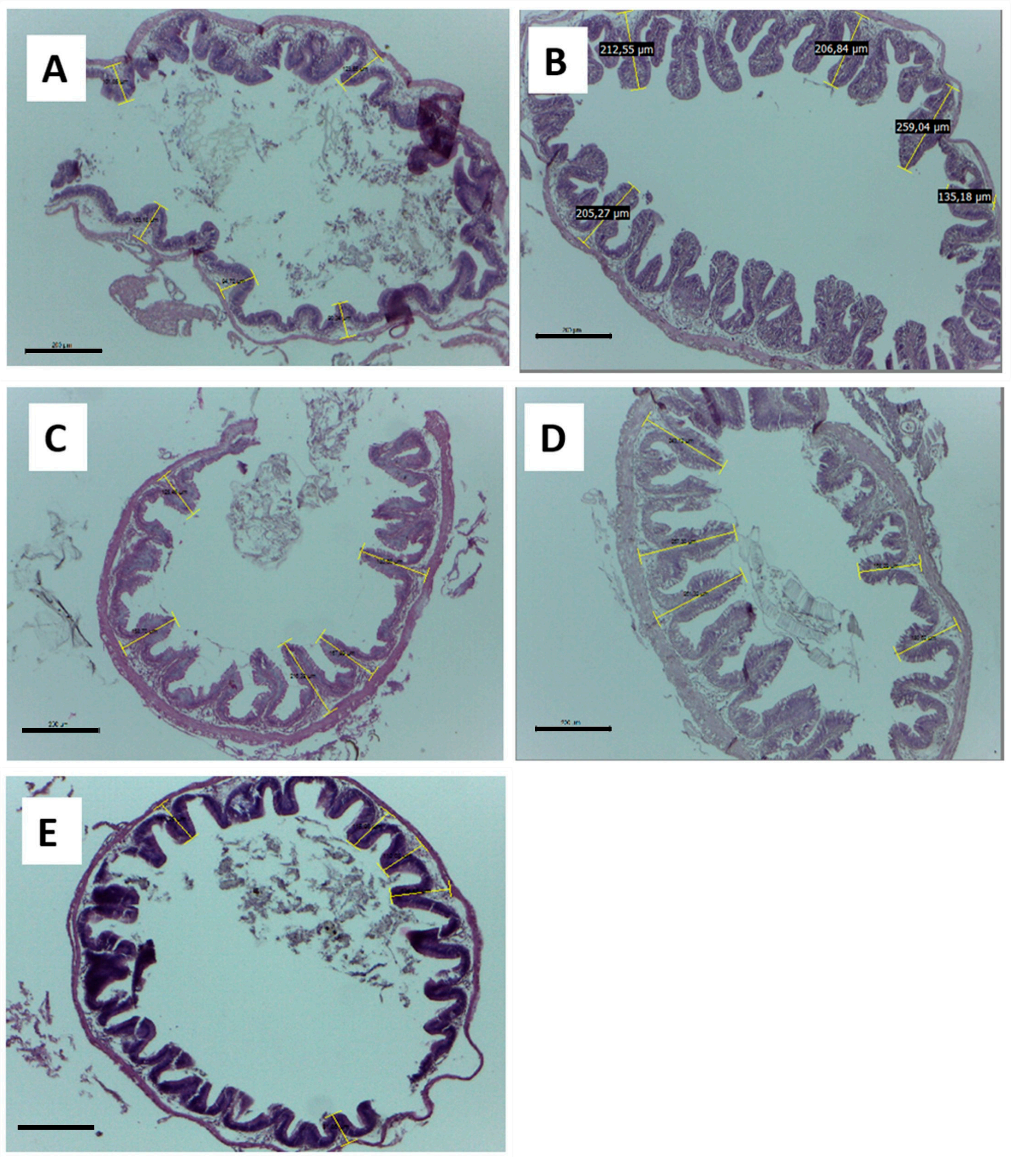

Figure S1. Intestinal micrographs of juvenile Nile tilapia (*Oreochromis niloticus*) fed experimental diets with (A) 0%, (B) 10%, (C) 20%, (D) 30%, (E) 40% *Limnospira maxima* biomass. The height of intestinal villi is highlighted in yellow. The scale bars represent 200 µm.

 https://doi.org/10.3390/ani16060889

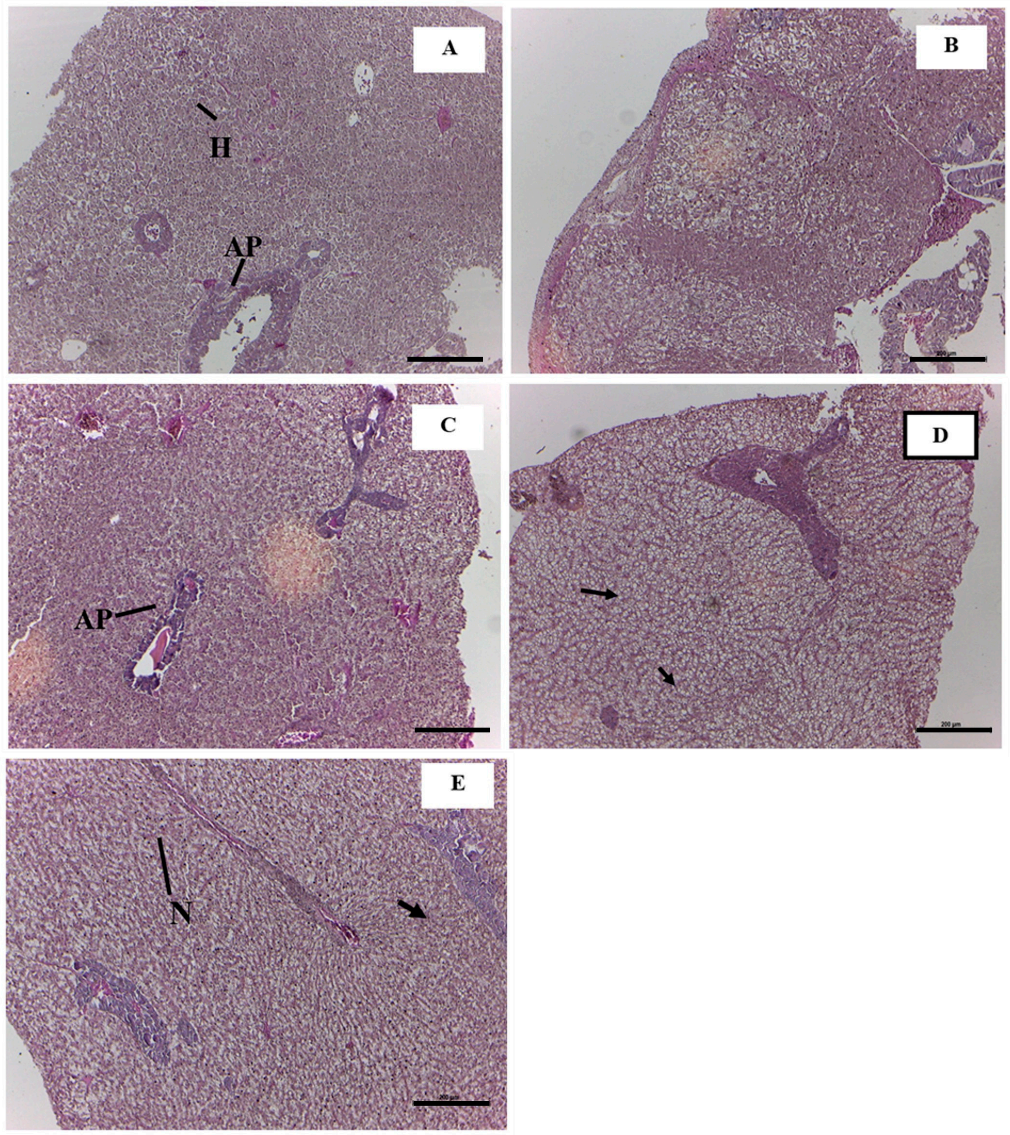

Figure S2. Liver micrographs of juvenile Nile tilapia (*Oreochromis niloticus*) fed experimental diets with (A) 0%, (B) 10%, (C) 20%, (D) 30%, (E) 40% *Limnospira maxima* biomass. The arrows indicate vacuoles, AP = pancreatic acini, and H = hepatocyte. The scale bars represent 200 μm.

Supplementary Tables

Table S1. Fatty acid profile of *Limnospira maxima* dry biomass used for experimental diets formulations. Data previously published in Araújo et al. (2024) [18]. Reproduced with permission from the publisher.

Fatty acid	Content (%)
Saturated fatty acids (SFAs)	
C16:0 (palmitic acid)	55.3 ± 4.54
C18:0 (stearic acid)	2.09 ± 0.41
Total SFAs	57.39
Monounsaturated fatty acids (MUFAs)	
C16:1*n*-7 (palmitoleic acid)	2.14 ± 0.24
C18:1*n*-9 (oleic acid)	5.98 ± 0.70
Total MUFAs	8.12
Polyunsaturated fatty acids (PUFAs)	
C18:2*n*-6 (linoleic acid)	4.78 ± 0.54
C18:3*n*-6 (γ-linolenic acid, GLA)	3.37 ± 0.41
C18:3*n*-3 (α-linolenic acid, ALA)	1.34 ± 0.11
Total PUFAs	9.49

The data represent the mean ± standard deviation of three independent experiments. The contents of annotated fatty acids correspond to 75% of the total fatty acid methyl ester (FAME) peak area; the remaining 25% correspond to unidentified FAME peaks.

Disclaimer/Publisher's Note: The statements, opinions and data contained in all publications are solely those of the individual author(s) and contributor(s) and not of MDPI and/or the editor(s). MDPI and/or the editor(s) disclaim responsibility for any injury to people or property resulting from any ideas, methods, instructions or products referred to in the content.
